# Primary anorectal malignant melanoma: A case report

**DOI:** 10.1097/MD.0000000000019028

**Published:** 2020-01-31

**Authors:** Xingdong Xu, Ting Ge, Gang Wang

**Affiliations:** a Department of General Surgery; b Department of Operating Room, The People's Hospital of China Three Gorges University, Yichang, Hubei 443000, China.

**Keywords:** abdominoperineal resection, anorectal malignant melanoma, metastases, prognosis, wide local resection

## Abstract

**Introduction:**

Anorectal malignant melanoma (AMM) is a rare and aggressive malignance with poor prognosis, yet no consensus of treatment exists to date. Abdominoperineal resection surgery (APR) is the standard treatment of anorectal malignant melanoma, capable of controlling lymphatic spread and obtaining a large negative margin for local control but it can lead to complications. Wide local excision (WLE) allows for quicker recovery and has minimal impact on bowel function (i.e., bypassing the need for a stoma).

**Patient concerns:**

A 66-year-old male patient presented with a 2-months history of painless rectal bleeding.

**Diagnosis:**

The characteristic finding from colonoscopy and magnetic resonance imaging led to a diagnosis of colorectal cancer. Immunohistochemistry analyses confirmed malignant melanoma. The tumor was classified as: HMB-45(+), S-100(+), CD117(±), PCK(−), ki-67(+, 10%).

**Interventions:**

The patient underwent abdominoperineal resection with no other adjuvant therapy.

**Outcomes:**

The patient is doing well at 24 month after the operation, with no signs of recurrence.

**Conclusion:**

AMM is a rare malignance, and is easy to misdiagnose. The therapy approach remains controversial. Every effort should be made to ensure prompt diagnosis and to define the optimally effective standard therapy approach.

## 1 Introduction

Primary anorectal malignant melanoma (AMM) is a rare and aggressive tumor, accounting for only 0.05% to 4.6% of all reported anorectal malignancies.^[1,2]^ The most common symptom prompting presentation of these patients is bleeding.^[3,4]^ Other symptoms include mass, pain, obstipation, and diarrhea, but pathologic diagnosis after a hemorrhoidectomy is not infrequent.^[1,3,4]^

There is currently no standard treatment for AMM, largely due to the overall low incidence and lack of evidence in the literature. While the typical therapeutic approach remains surgical resection, it is controversial whether abdominoperineal resection (APR) of the anorectum or wide local excision (WLE) provides the better outcome.

In this report, we present a patient who underwent APR for primary AMM and completed 24-months of follow-up.

## 2 Case report

**Figure 1:**
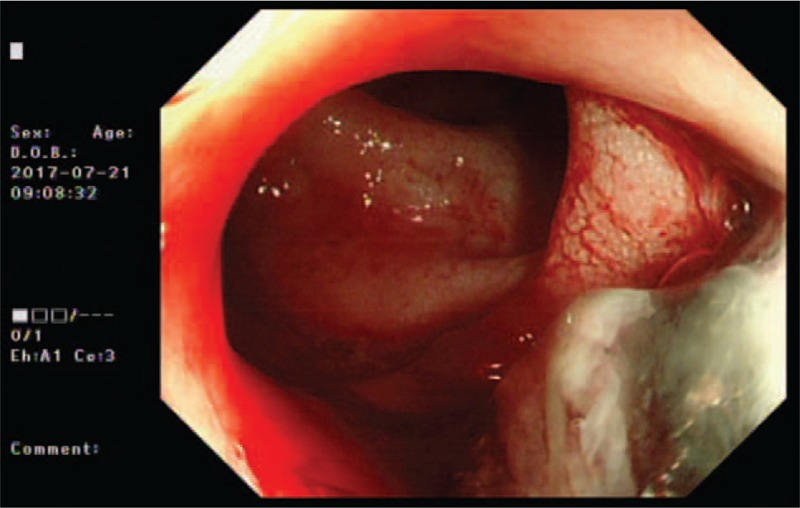
Images from the colonoscopy showing a tumor on the rectum. Colonoscopy revealed a mass at the rectum, with visual erosion and hemorrhage.

A 66-year-old man with complaint of painless rectal bleeding for 2 months was referred to the General Surgery Department of our Hospital. The patient presented in good condition, with no weight loss and reporting no significant past medical history. Rectal examination revealed an irregular mass near the anal verge. Colonoscopy showed a 30-mm lesion on the posterior wall of the rectum, located within 2 cm of the anal verge, and with visual erosion and hemorrhage (Fig. 1). Biopsies were taken for analysis.

**Figure 2:**
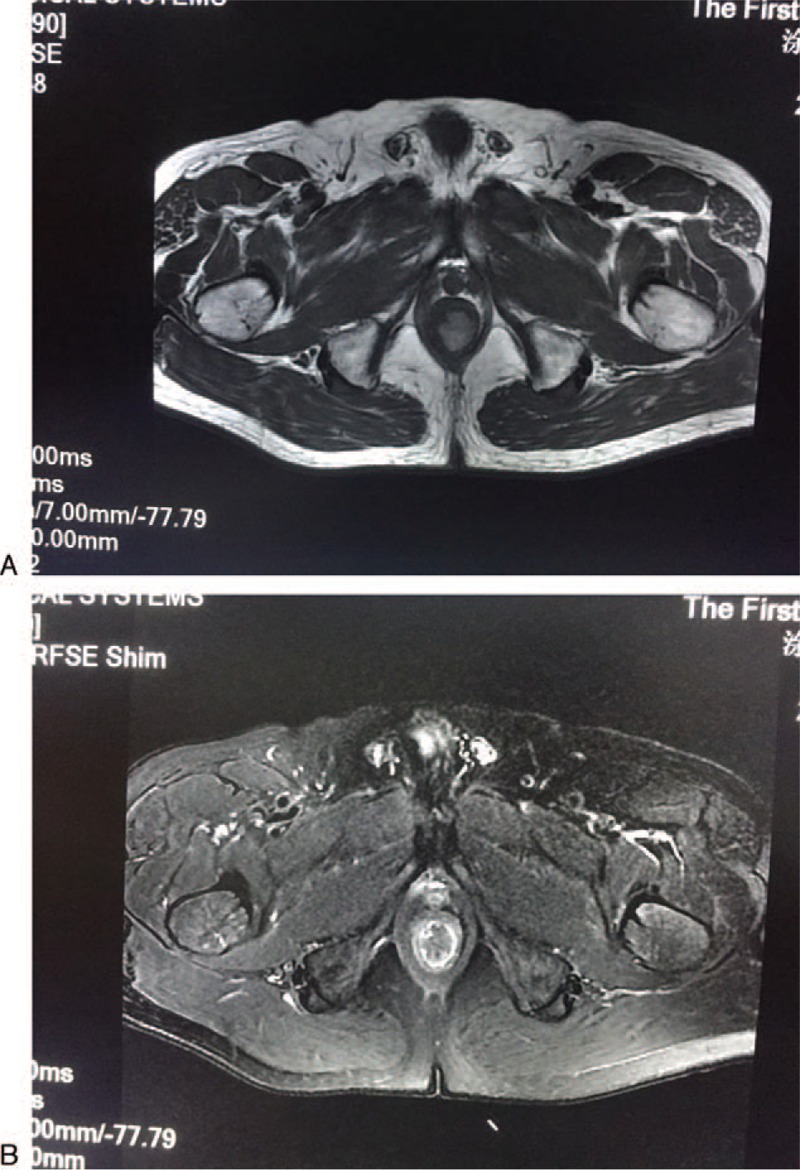
Magnetic resonance imaging showing a mass in the anal region. A: The tumors yielded high signal intensity on T1-weighted axial image; B: Low intensity was produced on the T2-weighted axial image.

**Figure 3:**
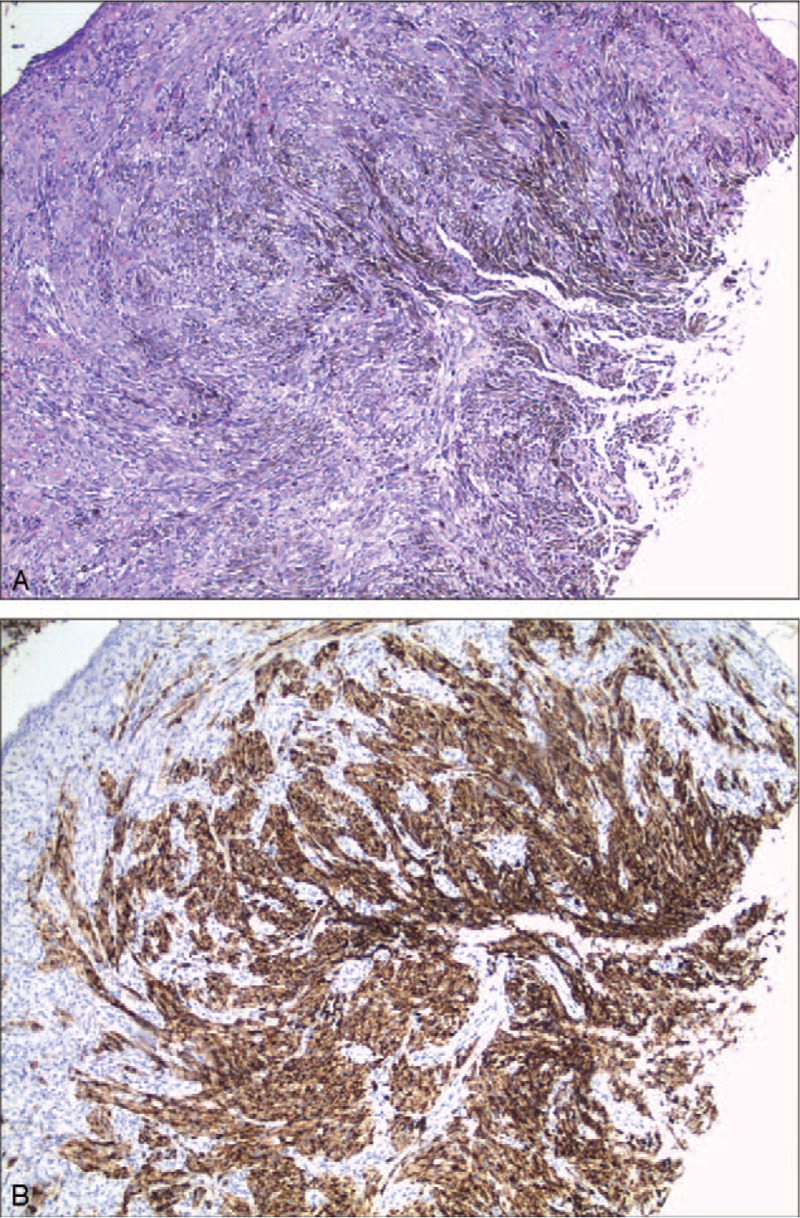
Histological analyses of biopsied tissues. A: Nested distributed tumor cells are shown full of melanin pigmentation in the cytoplasm (hematoxylin-eosin staining, 400×); B: Tumor cells showed positive staining for S-100 (immunohistochemistry staining, 400×).

Blood tests showed no blood count or biochemical abnormalities. Carcinoembryonic antigen and carbohydrate antigen 19–9 levels were also within normal ranges. Magnetic resonance imaging (MRI) detected a well-defined mass in the anal region (17.4 mm × 20 mm × 24 mm) (Fig. 2). Contrast-enhanced computed tomography detected no lymph node or distant metastasis. Histologic examination of the biopsied tissues showed nested distributed tumor cells with spindle-shaped nuclei, full of melanin pigmentation in the cytoplasm. The diagnosis of AMM was made. Immunohistochemistry analyses were also performed using specific markers to confirm malignant melanoma; the tumor was classified as HMB-45(+), S-100(+), CD117(±), PCK(−), ki-67(+, 10%) (Fig. 3).

The patient underwent APR. On intraoperative exploration, no distant organ metastasis was found. The mass is located in the posterior wall of the rectum, with the lower pole near the dentate line. The histologic examination of the operative specimen showed the tumor invaded through the muscularis propria. The 2 regional lymph nodes examined were free of tumor. Postoperative paraffin pathology of the resected tissues confirmed the diagnosis of stage 1 AMM. Postoperative adjuvant chemotherapy and radiotherapy was not performed because of the patient refused. Currently, at 2-year postsurgery, the patient is relapse-free and attends follow-up every 3 months on an out-patient basis.

## 3 Discussion

AMM is a rare type of melanoma, with a poor prognosis. The incidence of AMM increases with advanced age in both sexes and at all tumor sites. In general, the 5-year survival rate is low, being only 6% to 22% reportedly with a median survival period of 12.2 to 22 months.^[1–3,5–8]^

AMM lacks subjective symptoms in the early stage. The most common symptom is bleeding, with 53% to 89% of patients reporting this as the predominant complaint. The other symptoms include altered bowel habits, constipation, decreased stool caliber, unintentional weight loss, and palpable inguinal mass. Unfortunately, quite a few patients are diagnosed with AMM when they have already developed distant or regional metastasis.^[9]^ Weinstock reported that 41% of anorectal melanoma cases had regional spread and 22% had distant metastasis, while 37% had confined disease.^[10]^ The major sites of distant metastases are lung, liver, brain, bone, and breast.^[11–13]^ The patient presented herein did not experience any bleeding and had no distant or regional metastasis.

Histopathologically, AMM show considerable variability regarding the size and type of cells. They can be misdiagnosed as malignant lymphoma, small round cell sarcoma, spindle cell sarcoma, gastrointestinal stromal tumour, and epidermoid carcinoma. Thus, immunohistochemical analysis plays a pivotal role in the diagnosis of AMM. The most commonly used IHC stain in the diagnosis of AMM is Anti-S-100 protein and it is highly sensitive for melanocytic differentiation. Also, human melanoma black (HMB-45), Vimentin, and Melan A antibody are the melanocyte specific stains used for diagnosis of malignant melanoma.

The treatment of anorectal melanoma is controversial. While the typical therapeutic approach remains surgical resection, there is no consensus on which surgical approach –WLE or APR – is preferred. APR is regarded as the standard surgery for treatment of AMM because it can control lymphatic spread and obtain a larger negative margin for local control.^[14,15]^ However, APR can lead to complications such as urinary and sexual dysfunctions. Some investigators have recommended the WLE, instead, as it allows for quicker recovery and has minimal impact on bowel function (i.e., bypassing the need for a stoma).^[2,16]^ Evidence in the literature has suggested no significant statistical difference in 5-year survival rates and patterns of relapse between APR with a sufficient safety margin and WLE.^[17]^ Although, it is important to consider that WLE has no benefits when the tumor is invading the sphincter complex or causing chronic bleeding or obstruction. As such, many authors continue to advocate for APR to be performed when margins of the local excision are positive or in the event of recurrence.

Bullard et al reported that AMM recurrence is unrelated to the initial surgical procedure.^[18]^ Locoregional recurrence of AMM occurs more frequently at the inguinal lymph nodes than at the pelvic lymph nodes.^[2]^ Neither APR nor WLE affect any of the inguinal lymph nodes, so they do not offer an advantage in controlling locoregional recurrence.^[19]^

The prognosis of AMM is closely connected with the tumor biology, presence of perineural invasion, and lymphovascular involvement.^[20]^ It is also relevant to the decision as to whether a patient will undergo surgical resection or not. Positron emission tomography/computed tomography is the most widely adopted modality for detecting perirectal lymph nodes and screening for distant metastasis to assess patient status for the options of curative surgery.^[21]^ The patient in the present study underwent APR without adjuvant therapy. To date, he has been attending follow-up every 3 months and no considerable changes in his condition have presented.

In summary, AMM is a rare malignancy with extremely low 5-year survival rate. Due to low incidence, the treatment for AMM has not been well studied. The typical therapeutic approach remains surgical resection, but the surgical approaches are still controversial. Large-scale prospective clinical trials are needed to establish effective therapeutic approaches for AMM treatment.

## Acknowledgments

On behalf of all the authors, I acknowledge the contribution of Dr. Shenghua Cao to this article.

## Author contributions

**Data curation:** Xingdong Xu, Ting Ge.

**Writing – review & editing:** Gang Wang.
